# Genomic Tools for Medicinal Properties of Goat Milk for Cosmetic and Health Benefits: A Narrative Review

**DOI:** 10.3390/ijms26030893

**Published:** 2025-01-22

**Authors:** Keabetswe T. Ncube, Mamokoma C. Modiba, Takalani J. Mpofu, Khathutshelo A. Nephawe, Bohani Mtileni

**Affiliations:** Department of Animal Sciences, Tshwane University of Technology, Pretoria 0002, South Africamtilenib@tut.ac.za (B.M.)

**Keywords:** goat milk genomics, medicinal properties, eczema, psoriasis, bioactive compounds

## Abstract

Goat milk has gained recognition for its medicinal, cosmetic, and health benefits, particularly its potential to improve human skin conditions. Its therapeutic properties are attributed to bioactive compounds influenced by genes such as *lactoferrin* (*LTF*), *lysozyme* (*LYZ*), and *β-casein* (*CSN2*), known for their antimicrobial, immunomodulatory, and anti-inflammatory effects. Genetic factors are hypothesized to shape goat milk’s composition and its effectiveness in managing dermatological conditions like eczema and psoriasis. Understanding these genetic determinants is critical to optimizing the use of goat milk in skin health applications. This review aims to explore the application of genomic tools to elucidate the medicinal properties of goat milk and its implications for skin care. By identifying the specific genes and molecular mechanisms underpinning its therapeutic effects, genomic studies have provided insights into the bioactive constituents of goat milk, such as peptides, proteins, and lipids, which contribute to its dermatological efficacy. Candidate genes, including *growth hormone receptor* (*GHR*), *butyrophilin* (*BTN1A1*), and *lactoglobulin* (*LGB*), have been identified as critical for enhancing milk quality and functionality. Future research should integrate genomic data with functional studies to further investigate goat milk’s immunomodulatory, antimicrobial, and antioxidant activities. Such insights could advance targeted breeding strategies and innovative formulations for managing inflammatory skin conditions and promoting skin health.

## 1. Introduction

In the intricate tapestry of agricultural and human history, goats (*Capra hircus*) have emerged as steadfast companions, which led to their domestication and modification approximately 10,500 years ago in the Fertile Crescent [1]. Goat farming is a good option to support agricultural activities, socially and economically, due to their high adaptability to diverse environments and the inclusion of technical management to achieve maximum productivity [2]. With their primary role being meat, they are also used for milk, skins, and cashmere, providing sustenance to communities across the globe [3]. Milk from goats can also be used for a diverse range of dairy products, including butter, ice cream, cheese, buttermilk, condensed milk, yoghurt, flavored milk, sweets, and candies [4].

The global goat population exceeds 1 billion, with South Africa contributing approximately 7.8 million goats, over 63% of which are reared by small-scale farmers [3,5,6,7]. The South African dairy goat sector comprises around 4000 goats, producing approximately 1.4 million tons of milk annually [8]. European breeds (Saanen, Toggenburg, and Alpine) are renowned for their high milk yields, adaptability to temperate climates, and contributions to global dairy goat improvement [3,5,6,7]. In this review, we focus on the South African dairy goat breeds introduced in the 20th century from Europe, which have since adapted well to the country’s diverse and challenging conditions, demonstrating resilience to local diseases and suitability for milk production; the current production systems feature both purebred and crossbred Saanen, Toggenburg, and British Alpine goats derived from these initial imports [8,9,10].

The growing interest in preserving dairy goat breeds has prompted breeders to investigate the adequacy of the genetic diversity within the population to support the expanding industry [8]. In addition to genetic diversity, essential economic traits such as milk quantity, protein content, and milk fat remain pivotal. However, knowledge regarding the specific genomic loci governing these traits in goats is currently limited [11,12]. Comprehensive genomic insights into these loci would enhance our understanding of the genes and molecular mechanisms influencing goat milk composition, particularly those contributing to its therapeutic properties.

Goat milk and other goat-derived products contain numerous bioactive compounds that are promising for managing chronic diseases and skin conditions such as eczema and psoriasis [13,14]. The presence of peptides, fats, and oligosaccharides in goat milk suggests potential therapeutic applications in metabolic disorders and other diseases [14]. Nonetheless, a significant gap exists in the genomic understanding of the therapeutic attributes of goat milk, hindering the advancement of innovative applications in cosmetics and medicine. The genetic determinants that influence milk composition and its interactions with the cutaneous tissues remain inadequately understood. Given the formidable challenges posed by dermatological conditions like eczema and psoriasis, comprehensive insight into the metabolomic properties of goat milk is imperative. Thus, there is a critical need to unravel the genomic complexities inherent to goat milk to fully exploit its efficacy in addressing dermatological health concerns.

The historical recognition of goat milk in skincare highlights the need for deeper genomic studies to validate its therapeutic efficacy and expand its use in cosmetics and dermatology. Rich in proteins like casein and immunoglobulins, goat milk exhibits antibacterial, antioxidant, and immunomodulatory properties, enhancing skin health and offering various cosmetic benefits [14]. While anecdotal evidence suggests potential effectiveness in managing conditions such as eczema and psoriasis, the scientific understanding of goat milk’s genomic basis and molecular mechanisms is currently limited. Therefore, further research is imperative to elucidate these aspects comprehensively and unlock the full potential of goat milk for dermatological applications in a rigorous and evidence-based manner [14].

This review aims to explore the use of genomic tools to understand the medicinal properties of goat milk and its potential for skincare applications. It highlights the therapeutic value of goat milk in managing dermatological conditions such as eczema and psoriasis, presenting opportunities for sustainable skincare innovations. Analyzing the genomic and medicinal potential of goat milk is essential for improving milk production and developing targeted therapies. Advanced techniques like genome-wide SNP genotyping and milk protein sequencing are crucial for identifying the genetic mechanisms underlying goat milk’s bioactive properties. These approaches provide insights into SNPs, gene functions, and interactions between milk proteins and skin, which are key to therapeutic progress. The review emphasizes the genetic factors influencing goat milk composition and their implications for skin health, focusing on the molecular pathways and genetic determinants that are critical to dermatological treatments.

## 2. Benefits and Medicinal Properties of Goat Milk and Its Potential Use in Skin Therapies

**Figure 1:**
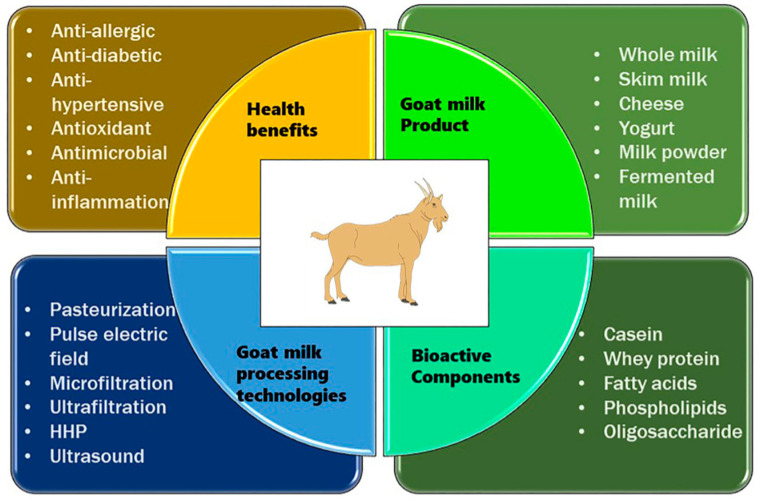
Processing, products, bioactive compounds, and health benefits of goat milk. Adopted from [20]. HPP = High-Pressure Processing.

Goat milk, a notable dairy alternative and a valuable source of essential nutrients, bioactive compounds, and potential health benefits, has gained scientific attention for its unique composition. Compared to cow milk, goat milk exhibits unique characteristics, such as smaller fat globules and different protein compositions, which contribute to its distinctive properties [15]. Several studies have demonstrated that goat milk is rich in essential nutrients, including calcium, phosphorus, potassium, vitamin A, vitamin B2 (riboflavin), and vitamin D, with potential implications for bone health, immune function, and overall well-being [16,17,18]. Additionally, the bioactive components present in goat milk, such as oligosaccharides and fatty acids, have been studied for their potential anti-inflammatory and immunomodulatory effects [18]. The therapeutic and hypoallergenic properties of goat milk, attributed to its reduced alpha-S1 casein and bioactive milk components, make it a promising alternative for individuals with cow milk allergies and of significant importance in human health and medical applications [19,20]. This highlights the scientific significance of goat milk and advances further exploration into its physiological effects and therapeutic applications. Goat milk offers several health benefits compared to milk from cows and other species (Supplementary Table S1), making it a valuable dietary option for many individuals. Figure 1 shows a detailed description of the processing of goat milk, its bioactive compounds, and its health benefits to better illuminate the understanding of goat milk, its products, and its benefits.

Goat milk is renowned for its high biological potential, and due to its natural origin and non-toxicity, it has multiple applications in the cosmetic and dermatological fields. These natural products are particularly rich in proteins such as casein, β-lactoglobulin, α-lactalbumin, lactoferrin, immunoglobulins, lactoperoxidase, lysozyme, and growth factors, all of which contribute to its diverse biological activities. These include antibacterial, antifungal, antiviral, anticancer, antioxidant, and immunomodulatory effects, among others [14]. Dairy derivatives, including those from goat milk, have found significant use in dermatological treatments, promoting wound healing, stimulating tissue regeneration, and managing skin conditions such as acne vulgaris and plaque psoriasis [14,21]. These products are particularly valued for their ability to improve skin health by reducing acne lesions, preventing blackheads, regulating sebum production, and alleviating inflammation. Additional benefits include moisturization, protection, toning, smoothing, anti-irritation, whitening, soothing, and anti-ageing effects [14]. While milk is primarily recognized as a raw material in the food industry, its substantial biological potential also drives its widespread use in the pharmaceutical and cosmetic sectors. Detailed analyses of its components and their therapeutic properties continue to substantiate its diverse roles in these industries [14].

Eczema and psoriasis are pervasive dermatological conditions characterized by chronic inflammation, compromised skin barrier function, and immune dysregulation, necessitating holistic and sustainable therapeutic solutions [14,22,23]. Goat milk has shown promise in promoting skin health due to its rich bioactive profile, including lactoferrin and lysozyme, which exhibit antimicrobial, anti-inflammatory, and immunomodulatory properties [14,22,23]. Preliminary evidence suggests that goat milk may alleviate symptoms by enhancing skin hydration, reducing inflammation, and improving barrier repair, with its low αS1-casein levels making it suitable for sensitive skin [14,16,23]. While much of the evidence remains anecdotal, studies have highlighted its ability to reduce *Staphylococcus aureus* colonization, a common eczema trigger, and modulate immune responses, supporting its potential as a natural remedy for eczema and psoriasis [21].

From its anti-inflammatory and antimicrobial activities to its moisturizing and antioxidant effects, goat milk offers a holistic approach to managing these chronic skin conditions. Further research is warranted to unravel the specific mechanisms of action underlying goat milk’s efficacy in skin therapy and to optimize its formulation and delivery for clinical applications, including the integration of goat milk genomics and delivery of goat-milk-based skincare products.

Goat milk is rich in bioactive components that have demonstrated significant anti-inflammatory properties. The anti-inflammatory effects of goat milk are primarily attributed to specific oligosaccharides and fatty acids that modulate immune responses and reduce the production of inflammatory cytokines. Studies have highlighted how these bioactive compounds can mitigate the underlying inflammation associated with eczema and psoriasis, thus contributing to symptom relief and improved skin health [22,24]. The ability of goat milk to modulate immune responses is particularly beneficial in chronic inflammatory conditions where the immune system plays a central role in disease progression. Furthermore, the lipid profile of goat milk, characterized by a high content of medium-chain fatty acids and triglycerides, provides excellent moisturizing and emollient properties. These lipids form a protective barrier on the skin’s surface, preventing transepidermal water loss and enhancing skin hydration. The significance of this barrier-forming ability has been emphasized [25], particularly for individuals with eczema and psoriasis, whose skin barrier function is often compromised. Goat-milk-based skincare products, therefore, offer effective moisturization and relief from dryness and itching. The protective barrier not only retains moisture but also shields the skin from environmental irritants, which can exacerbate skin conditions.

Studies indicate that the antimicrobial activity of goat milk components inhibits the growth of pathogenic microorganisms, such as *Staphylococcus aureus*, a common trigger for eczema exacerbations [23,26,27,28]. By preventing secondary infections and promoting wound healing, goat-milk-derived antimicrobial agents can significantly benefit individuals with compromised skin integrity. The presence of these antimicrobial compounds ensures that goat milk not only addresses inflammation but also the microbial infections that can complicate and worsen skin conditions.

In addition, goat milk contains a wealth of antioxidant compounds, including vitamins A, C, and E, as well as selenium and zinc. These antioxidants provide protective effects against oxidative-stress-induced skin damage, which is implicated in the pathogenesis of inflammatory skin disorders. Some studies suggest that the antioxidative properties of goat milk can mitigate oxidative damage, reduce inflammation, and support skin repair mechanisms [16,23]. By neutralizing free radicals, these antioxidants help to maintain skin health and prevent the exacerbation of skin conditions. The role of antioxidants is critical in skin therapy as oxidative stress is a key factor in skin ageing and inflammation.

Moreover, some genes have been associated with the medicinal properties of goat milk, and these pose great potential for the use of goat milk in skin therapies (Table 1). These genes encode proteins that play significant roles in enhancing skin barrier function, promoting wound healing, and regulating inflammatory and immune responses. Several candidate genes associated with the healing properties of goat milk, specifically in the context of treating skin conditions such as *lactoferrin* (*LTF*), *lysozyme* (*LYZ*), and *β-casein* (*CSN2*), that are known for their antimicrobial, immunomodulatory, and anti-inflammatory effects, respectively, have been identified. Understanding the genetic factors governing milk–skin interactions may provide valuable insights into the molecular pathways involved in healing skin conditions like eczema and psoriasis, ultimately contributing to the development of targeted goat-milk-based skincare products for maximum efficacy in management. The *LALBA* gene encodes the protein α-lactalbumin, which is pivotal in enhancing skin barrier function and promoting wound healing. α-lactalbumin is a key component of goat milk, contributing to its bioactive properties [29]. The protein helps in maintaining the integrity of the skin barrier, which is crucial for protecting against environmental insults and preventing transepidermal water loss, therefore promoting wound healing by facilitating the cellular processes involved in tissue repair. The research underscores the importance of α-lactalbumin in skin health, highlighting its potential therapeutic applications in skincare products designed for barrier repair and wound management [12,29,30].

The *LTF* gene, on the other hand, encodes lactoferrin, a multifunctional protein with significant anti-inflammatory and antimicrobial effects. *Lactoferrin*’s ability to bind and sequester iron limits the availability of this essential nutrient to pathogenic bacteria, thereby exerting broad-spectrum antimicrobial activity. Additionally, lactoferrin modulates immune responses, reducing inflammation and promoting a balanced immune environment. The dual role of *lactoferrin* in combating microbial infections and modulating inflammation has been demonstrated [16], further positioning it as a valuable component in therapeutic formulations aimed at treating skin infections and inflammation.

The *IL10* gene encodes interleukin-10, a cytokine with potent anti-inflammatory properties. Interleukin-10 suppresses the inflammatory response by inhibiting the synthesis of pro-inflammatory cytokines and promoting the differentiation of regulatory T cells, thus maintaining immune homeostasis. The critical role of interleukin-10 in regulating immune responses and its therapeutic potential in inflammatory skin diseases have been highlighted in previous studies [31,32]. By reducing inflammation and promoting immune regulation, interleukin-10 can alleviate the symptoms associated with chronic inflammatory skin conditions, enhancing skin health and recovery further, making it a potential candidate gene for the development of skin therapies. In addition, the *TGF-β1* gene encodes transforming growth factor-beta 1, a cytokine that modulates inflammation and promotes tissue repair. *TGF-β1* plays a pivotal role in regulating immune responses, facilitating tissue regeneration, and promoting wound healing. The multifunctional role of *TGF-β1* in skin health has been greatly discussed, therefore emphasizing its importance in managing skin injuries and chronic inflammatory conditions [13,33]. *TGF-β1*’s ability to promote tissue repair while modulating the inflammatory response makes it a key target for therapeutic interventions in skin repair and regeneration. The *COL7A1* gene encodes the collagen type VII alpha 1 chain, a critical component of the extracellular matrix that anchors fibrils attaching the epidermis to the dermis, thus maintaining skin stability and resilience, promoting wound healing and tissue regeneration, and reducing the risk of chronic wound complications [14,34].

The scientific evidence, although limited, highlights its multifaceted benefits in dermatological applications, suggesting that goat milk could offer a natural and effective alternative to conventional treatments. The genes associated with its healing properties provide a molecular basis for its therapeutic potential. Future research should focus on optimizing the formulation and delivery of goat-milk-based products to maximize their clinical efficacy, offering relief and improved quality of life for individuals with inflammatory and infectious skin disorders

## 3. Goat Milk Genomics and Genomic Tools

Advances in the genomic era have shown that more can be explored and achieved in terms of using genomics to reveal and aid in the understanding of genetic mechanisms in various organisms. Approximately 271 goat genes are associated with economic traits such as milk, fiber, and meat production, disease resistance, reproduction, and growth in goats, where five genes were specifically associated with milk [35,36]. Understanding the genetic basis of milk traits is essential for dairy goat breeding programs aimed at improving milk production efficiency and dairy product quality. Due to the significant economic impact of goat dairy products, studies on the genes associated with milk yield and quality have received more attention. Some of the genes influencing meat quality and yield have also been reported to affect milk yield and content traits, indirectly influencing the quality of milk and its derived products [37]. For instance, the *Insulin-like growth Factor 1* gene (*IGF-1*), which is linked to body growth, development, and metabolism, has also been reported to play a role in regulating the expression of milk protein and fat [37,38].

Research into goat milk genomics has unveiled promising insights into candidate genes associated with bioactive compounds that confer health and cosmetic benefits. The application of genomic tools in this context offers a systematic approach to identifying and understanding the genetic basis underlying these bioactive compounds, thereby exploring their potential applications. Genomic studies have identified various candidate genes in goat milk that encode proteins involved in the synthesis and regulation of bioactive compounds such as peptides, fatty acids, and antioxidants. These compounds have demonstrated beneficial properties in human health, including antimicrobial, anti-inflammatory, and immunomodulatory effects [16]. Furthermore, genomic tools enable the elucidation of genetic variations within these candidate genes among different goat breeds, which may influence the production levels and composition of bioactive compounds in milk. For instance, genetic variations in genes encoding milk proteins and enzymes can impact milk yield, protein content, and the presence of specific bioactive peptides [12].

**Figure 2:**
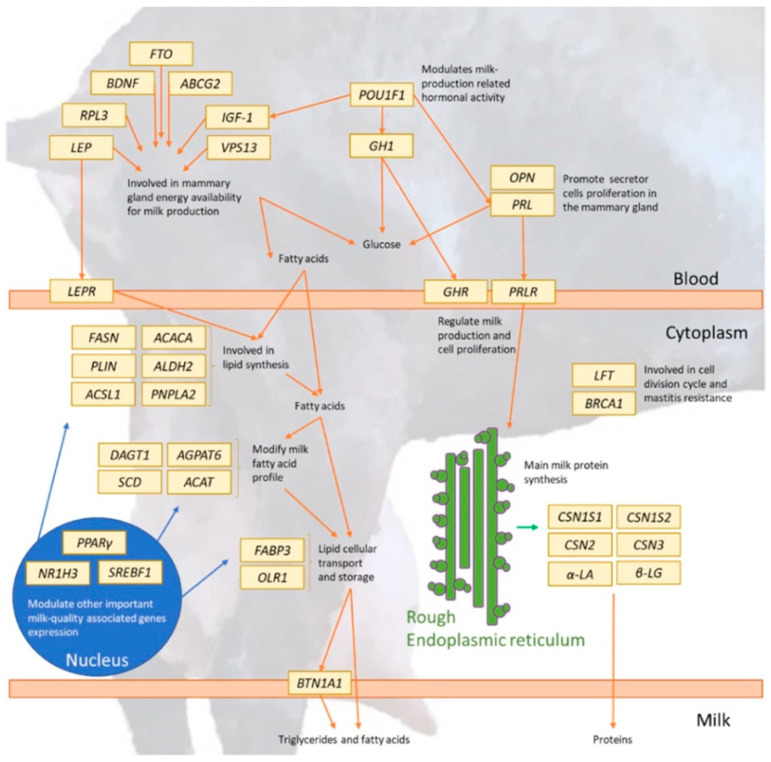
A representation of dairy goat candidate genes and their roles in milk production and quality [37].

In the cosmetics industry, bioactive compounds derived from goat milk, such as peptides and lipids, are increasingly recognized for their moisturizing, anti-ageing, and skin-barrier-enhancing properties [39]. Genomic approaches facilitate the identification of genes associated with these compounds, enhancing our ability to select and breed goats with desired traits for cosmetic applications. Table 2 and Figure 2 [37] show a representation of dairy goat candidate genes and their various roles.

Milk, proteins, and milk fat quantity are particularly important traits in dairy breed and milk production, and not much is known about the area of the genome that controls these important traits in goats. A comparative analysis of the signatures of selection for milk traits revealed candidate genes for milk composition and yield such as ABCG2 and NCAM2 [11].

Some of these genes show potential for use in optimizing milk quality for production purposes. For example, the *growth hormone 1* gene (*GH1*) has demonstrated its usefulness in stimulating udder development in transgenic goats and, therefore, increasing their milk yield [37]. Genetic studies investigating the healing properties of goat milk, particularly in the context of skin diseases, have shed light on the underlying mechanisms and identified the key genes associated with these beneficial effects. While the research in this area is still emerging, several studies have provided valuable insights into the genetic basis of goat milk’s therapeutic properties for skin health.

Through RNA-sequencing technology, the transcriptomic profile of goat mammary gland epithelial cells during lactation revealed the expression of genes involved in immune modulation, wound healing, and antimicrobial defense [42]. This study identified specific genes, such as *lactoferrin* (*LTF*) and *lysozyme* (*LYZ*), known for their antimicrobial and immunomodulatory properties, as being highly expressed in goat mammary epithelial cells, highlighting their potential role in conferring healing properties to goat milk [42]. While specific genetic studies directly linking genes to the healing properties of goat milk for skin diseases are limited, ongoing research in this area holds promise for uncovering novel genetic pathways and biomarkers associated with goat milk’s therapeutic effects. Further studies leveraging advanced genetic techniques, such as genome-wide association studies and transcriptomic analyses, may unravel the genetic mechanisms underlying goat milk’s healing properties and pave the way for the development of innovative therapies for skin diseases, and the next-generation genomic tools have proven to have great potential in this regard.

The next-generation era offers a range of tools that can generate high-quality data in a short time (www.illumina.com, accessed on 16 December 2024). These data can then be used to study informative genes that can assist in bringing about useful information and hence enable improved understanding of dairy goats, milk genetics and, more especially, the genes that are associated with the healing properties of goat milk. Hence, their potential can be ascertained and developed for their improvement. Several tools can be used in goat genomic studies. One such tool includes single-nucleotide polymorphisms (SNPs), which can explain most of the genetic discrepancies that are evident between animals [43]. Single-nucleotide polymorphisms (SNPs) are single-base-pair modifications within the genome [44] comprising non-synonymous and synonymous [45] as well as coding and non-coding variations. Coding SNPs are found near the coding regions of various genes, such as the mitochondrial DNA [46]. Next-generation sequencing (NGS) technologies have revolutionized the field of goat milk genomics by enabling high-throughput, cost-effective, and comprehensive analyses of the goat genome, transcriptome, and microbiome. NGS techniques, such as whole-genome sequencing (WGS), RNA sequencing (RNA-seq), and metagenomic sequencing, have been instrumental in advancing our understanding of the genetic basis of milk composition, quality, and bioactive properties in goats. Several techniques have been developed to study SNPs, which include SNP arrays, whole-genome sequencing (WGS), and targeted gene sequencing [45]. These technologies can be used to unravel the genomic potential of goat milk and its use for medicinal purposes.

### 3.1. The Illumina Goat SNP Chip

The progression of genetic technologies has instigated a surge of interest in genome-wide investigations [36]. The Illumina goat SNP50K is a high-density SNP chip consisting of markers evenly spaced across the goat genome and is a useful tool for population genetics, genetic diversity, and genomic association studies [47]. It was developed using six goat breeds and has been validated to be suitable for use in a variety of goat breeds [47,48,49]. This technology has been used in various studies in South African goats. One such study was performed on Angora goats, where the suitability of the Illumina goat SNP50K BeadChip for specific breeds was explored [48]. It has also been suggested [48] that the chip could be used as a tool for genome-wide association, genetic variation, signatures of selection, and genomic selection studies, as well as for parentage verification. The Illumina goat SNP50K chip was used in the analysis of the population structures of South African goat populations [50]. This study reported the feral Tankwa population to be a genetically distinct population, with the South African goat populations clustering according to their historical origins [50]. It further described the usefulness of the Illumina goat SNP50K in the investigation of the population structure, genetic diversity, and relationships between the SA non-descript goats and the feral Tankwa populations. The Illumina goat SNP50K chip also revealed valuable information on the demographic history of the South African goat populations [49]. The Illumina goat SNP50K BeadChip was used for body morphological traits in Sudanese goats [51].

Using this chip, a study on Canadian dairy goats revealed 189 unique and significant SNPs corresponding to 271 unique positional candidate genes within 50 kb upstream and downstream across breeds and traits [41]. This study further provides evidence for the economic importance of several candidate genes (e.g., *CSN1S1*, *CSN2*, *CSN1S2*, *CSN3*, *DGAT1*, and *ZNF16*) that have been associated with milk quality and production traits in the Canadian Alpine and Saanen populations [41]. In the past, the use of very few breeds (six) to develop the goat SNP50K [47] posed limitations in that the breeds that were used were not of African origin, and there may be some genetic variations that cannot be detected due to ascertainment bias [52]. While the BeadChip development initially focused on specific breeds, subsequent observations have revealed substantial genomic variations in excluded breeds, leading to broader applicability of the SNP50K BeadChip across various goat breeds [52] The latest iteration, Goat_IGGC_65K_v2, represents an updated version containing 59,727 SNPs, enhancing its genomic analysis capabilities [52], thus making it a suitable tool to use in unravelling the genomic potential of goat milk.

### 3.2. Next-Generation Sequencing (NGS)

Next-generation sequencing (NGS) is a high-throughput technology based on massively parallel sequencing, where millions of sequences are deciphered concurrently [53]. The technology originated in the early 2000s and demonstrated success through the complete sequencing of the human genome [53]. NGS consists of platforms that have proven to be highly quantitative and adaptive, with the promise to eliminate micro-array limitations [54]. It can identify transcriptomes without knowledge of a particular gene, therefore providing insights on alternative splicing and alternative variations in identified genes, and it also provides complete coverage in a relatively short period [54,55]. Platforms such as the Illumina HiSeq 2500 (Illumina Inc., San Diego, CA, USA) can produce about 500 Gb, and NovaSeq 6000 (Illumina Inc., San Diego, CA, USA) can produce about 3 TB of sequence data in one run in less than 10 days.

### 3.3. Whole-Genome Sequencing

Whole-genome sequencing (WGS) is a method whereby the whole genetic makeup of an organism or animal is sequenced (www.illumina.com, accessed on 16 January 2024). Whole-genome sequencing in practice does, however, not necessarily cover all the components of the genome (www.acmg.net, accessed on 16 January 2024). For significance, many sequence reads are needed (approximately 6.3 million), and multiplexing is also a challenge [56]. These aspects alone make the sequence costs per sample very high, thereby placing limitations on sample numbers [56].

WGS studies conducted on the South African feral Tankwa goat revealed variants associated with cellular pathways, defense, and immunity [57]. SNPs that show a potential role in goat growth performance, diseases, and adaptation processes have also been identified [57]. This technology has also been used in the identification of signatures of selection in important traits such as coat color, growth, reproduction, and high-altitude adaptation in goats [58,59].

The number of thoracic vertebrae is one of the important economic traits that influence carcass length and meat production in livestock, and WGS revealed candidate genes that were associated with the thoracic vertebrate number in sheep [60]. A study on indigenous Ethiopian goats identified the *retinoic acid receptor gamma* (*RARG*) gene to be of functional significance involved in the correct formation of the axial skeleton, including anteriorization of the cervical and thoracic vertebrae [61]. This gene has also been reported to play a significant role in production traits such as milk production, body size, and kidding in Liaoning cashmere goats [61]. Through WGS in Pakistan cattle breeds, it was revealed that most of the altered genes are significantly enriched in economically important biological processes such as heat tolerance, immune response, development, and sensory perceptions [62]. However, as mentioned above, the main limiting factor of this technology is the high cost per sample, making it too expensive, especially in the case of multiple samples.

Whole-genome sequencing has been very useful in the development of goat genomic studies, especially in the sequencing of goat reference genomes. The first draft genome achieved through WGS was from the female domestic goats Yunnan black goats with a genomic size of approximately 2.66 Gb [63]. This was followed by other goat genome sequences that have been successfully used in the identification of CNVs, SNPs, and other genomic variants [57].

### 3.4. RNA-Sequencing

RNA-seq is a high-throughput sequencing method for gene expression profiling that is widely used for mapping and quantifying transcriptomes and analyzing gene expression in various tissues [64], thus providing more accurate levels of those transcripts used to measure transcriptome composition and to discover new exons or genes [65]. RNA-seq is one powerful approach that is widely used. Transcriptomic studies have identified the genes that encode milk proteins, including caseins (e.g., *CSN1S1*, *CSN1S2*, *CSN2*, and *CSN3*) and whey proteins (e.g., *LALBA*, *LGB*, and *LTF*) [66,67]. These genes are highly expressed in mammary gland tissue during lactation and play crucial roles in determining milk protein composition and quality [67]. RNA-seq technology has also proven its usefulness in the gene expression profiling of intramuscular fat in Nellore cattle, where several genes associated with lipid metabolism and fatty acid composition were identified [68]. This demonstrates the potential for RNA-seq to be used as a tool for gene expression profiling as there are associations that can be linked to dairy goats’ traits and the identification of those genes associated with the healing properties of goat milk.

While specific research on the use of genomic tools for goat-milk-based therapies in eczema and psoriasis is limited, the existing evidence suggests promising avenues for further investigation. By elucidating the genetic basis of goat milk’s healing properties and understanding its interactions with skin biology, genomic research holds the potential to drive innovations in skincare and dermatological treatments.

With the lack of evidence on breed-specific medicinal properties of goat milk, it would be beneficial if researchers explored this area to investigate whether there are specific breed differences among the bioactive components associated with healing properties. To explore this, a comparative study design can be employed. This study would involve the collection of milk samples from various indigenous and commercial goat breeds under standardized feeding and management conditions to minimize confounding factors. A two-phase approach can be adopted: first, a biochemical analysis to identify and quantify bioactive compounds such as immunoglobulins, antimicrobial peptides, fatty acids, and antioxidants in the milk of each breed. Second, in vitro assays (e.g., antimicrobial, anti-inflammatory, and antioxidant tests) would be conducted to evaluate the functional efficacy of these compounds. To link the findings to potential medicinal benefits, cell culture models or animal trials could assess the milk’s effect on immune response, gut health, or wound healing. The data would then be analyzed to determine whether significant variations exist among the breeds, highlighting the most promising sources for medicinal applications. This interdisciplinary study would provide a robust foundation for promoting breed-specific utilization of goat milk in nutraceuticals and functional foods.

## 4. Conclusions and Recommendations

In conclusion, this review underscores the transformative role of genomic tools in elucidating the medicinal properties of goat milk, particularly its applications in managing dermatological conditions such as eczema and psoriasis. Goat milk is a rich source of bioactive compounds, including peptides, proteins, and lipids, which exhibit anti-inflammatory, antimicrobial, immunomodulatory, and antioxidant properties. These attributes make goat milk a promising natural therapeutic agent for inflammatory skin conditions. Genomic studies have identified the key genes contributing to these properties, including *LALBA* (*α-lactalbumin*), *LTF* (*lactoferrin*), *LYZ* (*lysozyme*), *IL10* (*interleukin-10*), *TGF-β1* (*transforming growth factor-beta 1*), and *COL7A1* (*collagen type VII alpha 1 chain*). These genes are directly associated with enhancing skin barrier function, modulating immune responses, promoting wound healing, and combating microbial infections. For instance, *LALBA* aids in skin barrier repair, *LTF* provides antimicrobial and anti-inflammatory effects, and *IL10* and *TGF-β1* regulate inflammation and tissue repair. *COL7A1* further supports skin integrity and regeneration, which is crucial for healing compromised skin affected by conditions like eczema and psoriasis.

The application of genomic tools, such as single-nucleotide polymorphism (SNP) genotyping and next-generation sequencing, has enabled researchers to identify genetic variations that influence milk composition and the bioactivity of its compounds. These advancements have facilitated targeted breeding strategies to optimize goat milk for skincare and medicinal use. By leveraging these genomic insights, goat milk can be tailored to address specific dermatological challenges and improve its therapeutic efficacy.

Future research should prioritize breed-specific genomic studies to explore variations in bioactive compound profiles across goat populations, identifying breeds with enhanced therapeutic traits. Targeted breeding programs should focus on increasing the prevalence of those traits that are linked to the treatment of eczema and psoriasis, such as the production of immunomodulatory and antimicrobial proteins. Furthermore, the development of innovative customized skincare products using genomic insights could maximize goat milk’s therapeutic benefits. Collaborative efforts between genomic researchers, dermatologists, and the cosmetics industry will be essential to translate these findings into effective and commercially viable applications. By harnessing the bioactive properties of goat milk through genomic tools, significant advancements can be achieved in personalized medicine and sustainable solutions for dermatological conditions.

## Figures and Tables

**Table 1:** Genes associated with healing properties of goat milk for skin diseases.

Gene	Name	Function	Associated Healing Properties	References
*LALBA*	*α-Lactalbumin*	Encodes α-Lactalbumin protein	Enhances skin barrier function; promotes wound healing	[12,29,30]
*LTF*	*Lactoferrin*	Encodes Lactoferrin protein	Anti-inflammatory and antimicrobial effects	[16]
*IL10*	*Interleukin-10*	Encodes Interleukin-10	Suppresses inflammation; regulates immune response	[31,32]
*TGF-β1*	*Transforming Growth Factor Beta 1*	Modulates inflammation; promotes tissue repair	Plays a key role in regulating immune responses and promoting tissue regeneration	[13,33]
*COL7A1*	*Collagen Type VII alpha 1 chain*	Encodes Collagen Type VII alpha 1 chain	Promotes wound healing and tissue regeneration	[14,34]

**Table 2:** Dairy goat candidate genes for milk production/yield and their roles.

Gene	Name	Associated Traits	References
*ABCG2*	*ATP Binding Cassette Subfamily G Member 2*	Milk production and composition traits	[11]
*ADAMTS20*	*ADAM metallopeptidase with thrombospondin type 1 motif 20*	Milk production and its protein content	[40]
*CK Deoxycytidine kinase*	*CK Deoxycytidine kinase*	Milk production	[40,41]
*CSN1S1*	*αs1 Casein*	Protein and fat content	[12]
*DGAT1*	*Diacylglycerol O-Acyltransferase 1*	Fat content	[12]
*MOB1B OB kinase activator 1B*	*MOB1B OB kinase activator 1B*	Milk production	[40,41]
*NCAM2*	*Neural Cell Adhesion Molecule 2*	Fat, protein, and milk yield	[11]
*PDE9A*	*Phosphodiesterase 9A*	Protein content	[12]
*PLD2*	*Phospholipase D 2*	Protein yield	[12]
*RPL8 Ribosomal protein L8*	*RPL8 Ribosomal protein L8*	Milk production	[40,41]

## Data Availability

The data that support the findings of this study are openly available from Preprint at https://www.preprints.org/manuscript/202404.1662/v1, (accessed on 10 December 2024).

## Supplementary Materials

The following supporting information can be downloaded at: https://www.mdpi.com/article/10.3390/ijms26030893/s1.
