# Supplementary material for: Genomic Tools for Medicinal Properties of Goat Milk for Cosmetic and Health Benefits: A Narrative Review
Source: Int J Mol Sci. 2025 Jan 22;26(3):893. doi: 10.3390/ijms26030893 (PMC11817167; doi:10.3390/ijms26030893)
Supplement: Supplementary file 1 [file ijms-26-00893-s001.zip › ijms-3405089-supplementary.pdf]

Supplementary Table S1: A comparison of goat milk with other common dairy animal milk focuses on their nutritional composition, health benefits, and medicinal properties.

	Dairy Animals					
	**Goat**	**Cow**	**Sheep**	**Buffalo**	**Camel**	**Donkey**
**Protein Content**	3.2 g per 100 g; contains less αs1-casein, which may reduce allergenicity.	3.2 g per 100 g; higher in αs1-casein, a common allergen.	5.4 g per 100 g; rich in all essential amino acids.	4.5 g per 100 g; high in casein, beneficial for cheese production.	3.1 g per 100 g; lacks β-lactoglobulin, reducing allergenic potential.	1.5–1.8 g per 100 g; contains hypoallergenic proteins.
**Fat Content**	4.0–4.5 g per 100 g; smaller fat globules enhance digestibility.	3.3 g per 100 g; larger fat globules.	6.0 g per 100 g; high in medium-chain fatty acids.	7.4 g per 100 g; high in saturated fats.	3.5 g per 100 g; contains higher levels of unsaturated fatty acids.	0.5–1.8 g per 100 g; low fat, mostly unsaturated.
**Lactose Content**	4.6 g per 100 g; slightly lower than cow milk, potentially easier for lactose-intolerant individuals.	4.8 g per 100 g; standard reference for lactose content.	4.8 g per 100 g; similar to cow milk.	4.9 g per 100 g; similar to cow milk.	4.8 g per 100 g; lower lactose content, often better tolerated by lactose-intolerant individuals.	6.0–7.0 g per 100 g; higher lactose content; promotes gut health.

**Minerals**	Higher in calcium (0.13 g per 100 g), magnesium (0.014 g per 100 g), and phosphorus (0.11 g per 100 g) compared to cow milk.	Rich in calcium (0.12 g per 100 g) and phosphorus (0.10 g per 100 g); standard reference for mineral content.	Higher in calcium (0.17 g per 100 g) and phosphorus (0.13 g per 100 g); supports bone health.	Highest in calcium (0.19 g per 100 g) among common dairy milks; beneficial for bone strength.	Rich in potassium (0.15 g per 100 g) and magnesium (0.15 g per 100 g); supports cardiovascular health.	High in calcium (0.16 g per 100 g), magnesium, and zinc; promotes bone and immune health.
**Vitamin Content**	High in vitamin A (0.185 mg per 100 g), riboflavin (B2) (0.138 mg per 100 g), and niacin (B3) (0.277 mg per 100 g); supports vision and skin health.	High in vitamin B12 (0.45 μg per 100 g) and riboflavin (0.183 mg per 100 g); essential for red blood cell formation.	Rich in vitamins A (0.18 mg per 100 g), D (0.05 μg per 100 g), and E (0.12 mg per 100 g); supports immune function and skin health.	High in vitamin A (0.18 mg per 100 g); supports vision and immune function.	Rich in vitamin C (3.0 mg per 100 g); supports immune health.	Rich in vitamin C (3.0–7.0 mg per 100 g); enhances immunity.
**Digestibility**	Smaller fat globules and lower lactose content enhance digestibility; may be suitable for individuals with mild lactose intolerance.	Standard digestibility; may cause discomfort in lactose-intolerant individuals.	High fat and protein content; may be harder to digest for some individuals.	High fat content; can be challenging to digest for some individuals.	Contains hypoallergenic proteins and lower lactose; highly digestible, even for lactose-intolerant individuals.	Highly digestible due to low fat and unique protein structure.
**Health Benefits**	May reduce inflammation; supports gut health; beneficial for skin conditions like eczema and acne.	Supports bone health due to high calcium content; widely consumed.	High in bioactive peptides; may have antimicrobial properties.	High in energy; traditionally used in Ayurvedic medicine for stamina and strength.	Anti-inflammatory properties; may aid in managing diabetes and cardiovascular health.	Contains bioactive compounds with antimicrobial and antioxidant properties; may aid in gut health and skin conditions.

**Medicinal Benefits**	Contains bioactive compounds that aid in reducing cholesterol levels and improving gut microbiota; potential benefits for individuals with cow milk allergies.	May support bone health; potential allergen for some individuals.	Rich in immune-boosting components like zinc; supports muscle repair.	Provides high energy; used in traditional medicine systems.	Contains insulin-like proteins; may benefit glycaemic control in diabetics.	May support immune function, promote gut health, and aid in managing allergies and skin conditions.
